# The Impact of COVID-19 on Patient Safety: A Survey of Acute-Care Registered Nurses in New Jersey

**DOI:** 10.1017/ash.2021.105

**Published:** 2021-07-29

**Authors:** Monika Pogorzelska-Maziarz, Mary Lou Manning, Angela Gerolamo, Mary Johansen, Irina Grafova, Suzie Crincoli, Pamela de Cordova

## Abstract

**Background:** As the world grapples with the pandemic of severe acute respiratory syndrome coronavirus 2 (SARS-CoV-2), it is important to consider the full impact of coronavirus disease 2019 (COVID-19) on healthcare delivery. Evidence from outbreaks of novel H1N1 and Ebola indicates that response to these types of outbreaks requires extraordinary resources, which are diverted from routine infection prevention and control activities. However, little is known about the impact of COVID-19 on adherence to patient safety protocols in hospitals, including infection prevention and control activities. We have described the reports of acute-care registered nurses (RNs) in adhering to patient safety protocols while delivering care to COVID-19 patients. **Methods:** In October 2020, we conducted a cross-sectional electronic survey of all active RNs in the state of New Jersey who provided direct patient care in a New Jersey hospital in an emergency or adult inpatient unit during the onset of the COVID-19 pandemic. **Results:** More than 3,027 RNs participated in the survey, for a 15% response rate based on number of eligible RNs. Moreover, 15% of respondents reported that they tested positive for COVID-19 during the initial peak of COVID-19 in New Jersey (March–June 2020). Most RNs reported that the number of patients they were assigned during the first peak of the pandemic affected their ability to adhere to patient safety protocols (eg, deep-vein thrombosis screening, central-line bundles, pressure ulcer prevention). In open-ended responses, they shared that being understaffed, the extra time it took for downing and doffing of PPE, the lack of access to ancillary staff (ie nursing assistants, runners), and the need to cluster care affected the quality of care. A nurse working in the intensive care unit (ICU) lamented, “We were sometimes given 4–5 ICU patients who were very sick and required a lot of care. Shortcuts had to be taken to prioritize the most important needs. Sometimes IVs remained longer than desired. Foleys remained in longer. To avoid PPE shortages, we didn’t go into the rooms nearly as much as we normally would, [and] things got missed.” Feelings of being overwhelmed and helpless permeated the nurses’ comments. **Conclusions:** When caring for COVID-19 patients, frontline nurses struggled with adherence to necessary patient safety protocols, which ultimately disrupted care delivery. Future research should quantify the extent to which the COVID-19 pandemic affected care delivery, including adherence to patient safety protocols among frontline providers.

**Funding:** No

**Disclosures:** None

